# Selection and application of biological safety cabinets in diagnostic and research laboratories with special emphasis on COVID-19

**DOI:** 10.1063/5.0047716

**Published:** 2021-08-12

**Authors:** Shailesh D. Pawar, Ajay B. Khare, Sachin S. Keng, Sadhana S. Kode, Deeksha S. Tare, Dinesh K. Singh, Ramesh L. More, Jayati Mullick

**Affiliations:** 1 ICMR-National Institute of Virology, Microbial Containment Complex, 130/1, Sus Road, Pashan, Pune 411021, India; 2 ICMR-National Institute of Virology-Mumbai Unit (Formerly Enterovirus Research Center), Haffkine Institute Compound, Acharya Donde Marg, Parel, Mumbai 400 012, India

## Abstract

The ongoing coronavirus disease (COVID-19) pandemic is a global public health emergency. Adherence to biosafety practices is mandatory to protect the user as well as the environment, while handling infectious agents. A biological safety cabinet (BSC) is the most important equipment used in diagnostic and research laboratories in order to safeguard the product, the person, and the environment. The World Health Organization has emphasized the use of validated BSCs in order to ensure quality of the results. There are different classes of BSCs that are used in various work environments based on the need. It is imperative to use appropriate levels of biosafety and types of BSCs in laboratories based on the risk assessment of the pathogen used. During the development of COVID-19 laboratories and training of laboratory staff, we came across several queries about the functions and selection of BSCs and realized that the knowledge about the detailed information on selections and applications of BSCs is scanty. There are several guidelines regarding the biosafety aspects for diagnostic and research laboratories handling infectious pathogens from national and international agencies. However, there is no detailed information on the use of appropriate types of BSCs and their functions in the context of Severe Acute Respiratory Syndrome-Coronavirus 2 (SARS-CoV-2). In view of this, the present paper describes in detail the selection and applications of BSCs, which could be useful for laboratories handling or planning to handle SARS-CoV-2 and suspected samples.

## INTRODUCTION

Infectious diseases have been among the top ten causes of mortality in humans across the world (World Health Organization, 2018). The current pandemic of coronavirus disease 2019 (COVID-19) caused by the Severe Acute Respiratory Syndrome-Coronavirus 2 (SARS CoV-2) resulted in 167 423 479 confirmed cases worldwide, as of May 26, 2021 (World Health Organization, 2020a). The World Health Organization (WHO) guidelines for laboratories testing human samples suspected for COVID-19 recommend that testing of clinical specimens should be performed in appropriately equipped laboratories (World Health Organization, 2020b). The staff should be trained in biosafety and biosecurity practices as well as for handling respiratory samples for viral diagnosis (World Health Organization, 2020b). The WHO suggests that the national guidelines on laboratory biosafety should be followed by each nation under all circumstances (World Health Organization, 2021).

A biological safety cabinet (BSC) is the most important equipment for biocontainment used in diagnostic, microbiology, virology, research, and biosafety laboratories in order to safeguard the product, the person, and the environment. Laboratory activities, such as specimen separation, aliquoting, vortex-mixing, centrifugation, use of pipettes, and opening of pressurized containers, generate potentially infectious aerosols and microparticles. As BSCs act as the primary barrier and provide a safe work environment for the worker to handle high-risk pathogens such as SARS-CoV-2, certification of BSCs is of utmost necessity for their proper functioning. Certification of BSCs by an accredited agency based on systematic assessments and documentation would ensure that the equipment conforms to specified requirements as per the standard guidelines [World Health Organization (WHO), 2020c].

Organizations such as the National Accreditation Board for Testing and Calibration Laboratories (NABL), which is one of the signatories of the “International Laboratory Accreditation Cooperation” and the “Asia Pacific Laboratory Accreditation Cooperation,” provide accreditation of technical competence of testing, calibration, and medical laboratories (NABL, 2020). The Indian Council of Medical Research-National Institute of Virology (ICMR-NIV), Pune, is the only laboratory in India accredited by the NABL for the certification of BSCs.

The ongoing COVID-19 pandemic is a public health emergency, which created an urgent need to rapidly establish laboratories for the diagnosis of SARS-CoV-2 around the world. A BSC is critical equipment in these laboratories to handle infectious specimens. There are several guidelines regarding the biosafety aspects for diagnostic and research laboratories handling infectious pathogens from national and international agencies. However, there is no detailed information on the use of appropriate types of BSCs and their functions in the context of SARS-CoV-2. During the development of COVID-19 laboratories and training of laboratory staff, we came across several queries about the functions and selection of BSCs and realized that the knowledge about the detailed information on selections and applications of BSCs is scanty [World Health Organization (WHO), 2020c; 2020d]. In view of this, the present paper describes in detail the selection and applications of BSCs, which could be useful for laboratories handling or planning to handle SARS-CoV-2.

## BIOSAFETY CONSIDERATIONS AND COVID-19

In the current scenario of the COVID-19 pandemic, adherence to appropriate biosafety practices is pivotal in diagnostic and research laboratories. Biosafety is the application of the containment principles, technologies, and practices that are implemented to prevent unintentional exposure to biological agents or their inadvertent release [World Health Organization (WHO), 2020d]. Appropriate biosafety in a laboratory can be achieved through four controls. The *administrative controls* include management of laboratory functioning, training, and vaccination of the staff involved in testing activities; the *engineering control*s include the use of safety equipment such as BSCs as a primary containment and the construction and design of the facility; and the use of personal protective equipment (PPE) and adherence to standard operating procedures (SOPs). Application of all these four controls accounts for the optimum biosafety while working with infectious agents in a laboratory.

Following appropriate biosafety practices in virology laboratories enables researchers to work safely with high-risk pathogens. Risk assessment is the combination of identifying the hazard, risks, threats, procedures, facilities, personnel, etc. Assessment of the level of risk likely to be posed by an organism is crucial while choosing the level of the biosafety laboratory. The laboratories are classified as Biosafety Level (BSL)-1, BSL-2, BSL-3, and BSL-4, based on a composite of the design features, construction, containment facilities, equipment, PPE, practices, and operational procedures required for working with agents from various risk groups [World Health Organization (WHO), 2020d; CDC, 2020]. For the work on SARS CoV-2, the WHO recommends the use of a BSL-2 laboratory for non-propagative work and the use of a BSL-3 laboratory for propagative work (World Health Organization, 2021). The information on the infrastructure, the biosafety measures of testing laboratories providing COVID-19 diagnosis, and the associated biorisk to the individuals and the community have been reported (Mourya *et al.*, 2020; Rabi’u *et al.*, 2021).

Microorganisms have been classified into four categories based on factors such as pathogenicity, infectious doses, transmission modes, host range, availability of preventive measures, and effectiveness of treatment for the disease caused [World Health Organization (WHO), 2020d]. The SARS-CoV-2 virus has been classified as risk group 3 [Committee on Biological Agents (ABAS), 2020; Kaufer *et al*., 2020]. It is recommended that the specimens for molecular testing should be handled in BSL-2 or equivalent facilities (World Health Organization, 2021). However, a BSL-3 laboratory is recommended when the virus culture is being attempted or large quantities of virus are being handled, as the chances of generation of aerosols increase (World Health Organization, 2021). The processing of all specimens for further testing should be carried out in a validated BSC with proper PPE to ensure the safety of all (World Health Organization, 2021; Mourya *et al.*, 2020).

## BIOLOGICAL SAFETY CABINETS

The BSC is a chamber constructed of stainless steel. It has a front glass window of adjustable height, a ventilation system with an electrical motor, a ventilator, and a set of ducts, which while functioning generate an air pattern. The air enters the BSC through the front grill, and then, it is either exhausted or partially recirculated after filtration through High Efficiency Particulate Air (HEPA) filters. Internally, the air is passed through front and rear grills and ducts and is finally treated in HEPA filters. Broadly, the important parts of a BSC comprise a blower, the HEPA filter, the workspace, sash, a common duct, a front grill, and a rear grill. The BSC provides personal, product, and environmental protection. The airflow inside a BSC is maintained in such a way that no air escapes out from the workspace, thereby protecting the worker. Inside the BSC, HEPA-filtered sterile air is circulated over the samples ensuring that the samples being handled do not get cross-contaminated or contaminated by outside flora, affecting the results. In addition, the contaminated air from the workspace is not exhausted to the environment without being filtered through HEPA filters, thus conferring environmental protection.

## MECHANISM OF HIGH EFFICIENCY PARTICULATE AIR (HEPA) FILTRATION

HEPA is a type of pleated mechanical air filter that removes at least 99.97% of any airborne particles with a size of 0.3 *μ*m (United States Environmental Protection Agency, 2019). Considering the definition of HEPA filters, it could be misunderstood that the microorganisms of size below 0.3 *µ*m can easily pass through the filter, and the BSC would not be effective in handling such microorganisms. Therefore, understanding the construction of the filter is important in order to understand its function. The HEPA filters comprise randomly placed, compressed borosilicate fibers that are placed in a pleated fashion to increase the surface area and the life of the filter. These pleated media are then fixed into the frame. The air flows through this assembly. The contaminated air consists of particulate matter that can be classified into three groups based on their size as large, small, and microscopic. All three kinds of particulate matter travel in the air stream with different properties. The larger particles are heavier and follow a straight-line path in the air stream, due to which, when these particles pass through the HEPA filter, they collide with the fibers and get captured. This process is known as *impaction* in which particles larger than 0.5 *µ*m get arrested in the filter. The smaller particles in the air are lighter in nature and have the ability to change their direction and follow the air stream. These particles pass through the randomly placed borosilicate fibers along with the air stream, and whenever the gap between the air stream and the borosilicate fiber is smaller than the radius of the particle, it gets arrested. This phenomenon is called *interception*, due to which the particles of size 0.5–0.2 *µ*m are captured. The microscopic particles of size less than 0.3 *µ*m have the tendency to follow a random zig–zag path caused by Brownian motion. Due to this property, these particles cannot follow the air stream moving through the filter and get arrested into the fibers, which is called *diffusion*.

The combined effect of impaction, interception, and diffusion processes is that particles of all sizes whether higher or lower than 0.3 *µ*m are arrested in the HEPA filter (First, 1998). The particles of size 0.3 *µ*m are called the most penetrating particles. The efficiency of HEPA filters for 0.3 *µ*m sized particles is 99.97%, thereby enabling only 0.03 particles out of 100 to pass through.

This mechanism of HEPA filtration can effectively prevent the escape of SARS-CoV-2 from the BSC. It has been reported that SARS-CoV-2 is transmitted by aerosols and respiratory droplets (World Health Organization, 2020e). The diameter of the virion is 60–140 nm (Bar-On *et al.*, 2020). The diameter of a respiratory droplet is >5–10 *µ*m and that of aerosols is ≤5 *µ*m (World Health Organization, 2020e). Respiratory specimens such as nasal, throat swabs, and nasopharyngeal aspirates are the preferred specimens for the laboratory diagnosis of SARS-CoV-2. Handling of these specimens includes procedures such as pipetting, vortex-mixing, and aliquoting, which could potentially generate aerosols. Considering the size of aerosols generated during handling SARS-CoV-2 specimens and other procedures, the BSCs would protect the person and environment and prevent laboratory infections. Therefore, the use of appropriate BSCs is of utmost importance.

### Selection of BSCs for COVID-19 laboratories

BSCs are classified based on parameters such as the inflow velocity, the amount of air recirculated or exhausted, and the type of exhaust (World Health Organization (WHO), 2020d; CDC, 2020). There are three types of BSCs, namely, class I, class II (A1, A2, B1, and B2), and class III. The important parameters for the functioning of these BSCs are summarized in Table I.

**TABLE I.** Salient features of different classes of biological safety cabinets and their uses (From World Health Organization, *Laboratory Biosafety Manual*, 4th Edition. Copyright 2020 World Health Organization. Licence: CC BY-NC-SA 3.0 IGO and NSF NSF/ANSI 49-2020).

Parameters	Class I	Class II				Class III
		A1	A2	B1	B2	
Type of	Personnel and	Personnel, product, and environment				Personnel, product,
protection	environment					and environment
	Risk groups 1,	Risk groups 1, 2, 3, and 4 (if positive				Risk groups 1, 2, 3, and 4
Risk	2, 3, and 4 (if	pressure suit used)				
group	handled in suit					
	laboratory)					
Volatile	Yes	No	No	Yes	Yes	Yes
radionuclide/				(in		
chemical				small		
protection				quantity)		
Supply air	Not HEPA-filtered	HEPA-filtered				HEPA-filtered
Exhaust air	HEPA-filtered	HEPA-filtered				HEPA-filtered
Face velocity (m/s)	0.36	0.38	0.51	0.51	0.51	Not applicable
Airflow % exhausted	100	30	30	>50	100	100
Airflow % recirculated	0	70	70	<50	0	0
Exhaust	Hard	Room or thimble	Room or thimble	Hard	Hard	Hard
system	duct	connection	connection	duct	duct	duct

The selection of BSCs for the use in laboratories of different biosafety levels is based on the pathogen used and also on the risk assessment of the activities that are proposed to be carried out with the pathogen. In class I type BSCs, as room air directly enters the work surface without filtration through the HEPA filter, there would be no protection of the product. The class III BSC is a sealed BSC and is also called a “glove box” or an “isolator.” In these cabinets, work has to be performed through the attached gloves. These types of BSCs are used for cabinetry type BSL-4 laboratories for handling viruses such as avian influenza H5N1, Ebola, Nipah, and other exotic viruses. Class II A1, A2, and B1 BSCs have the feature of recirculation of air from the workspace after being HEPA-filtered. The class II B2 BSCs have 100% exhaust of the air from the workspace and, thus, are ideal to handle large amounts of radionuclides and volatile chemicals along with the microbiological agent (Table I). Similarly, the purpose of class II B1 BSCs is to handle small amounts of radionuclides and volatile chemicals along with the microbiological agent. The ventilation of the laboratory is maintained as per the risk assessment and requirements. The containment laboratories function under negative pressure. Whenever class A type BSCs are used, the ventilation system of the laboratory does not get disturbed. However, when hard-ducted B type BSCs are used, the room pressure and ventilation system get imbalanced due to the exhaust. These factors should be considered while installation and use of BSCs.

**FIG. 1.**
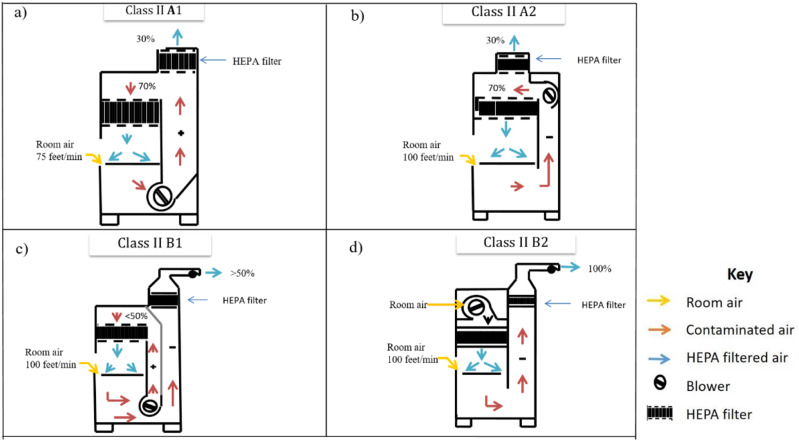
Comparison of class II Biological Safety Cabinets (BSCs). A schematic representation of the structural aspects of class II BSCs: (a) A1 BSC, (b) A2 BSC, (c) B1 BSC, and (d) B2 BSC has been shown. The yellow, orange, and blue arrows indicate the direction of the flow of room air, contaminated air, and filtered air, respectively, inside the biological safety cabinets. The key shows the notation used for the HEPA filter and blower.

For handling suspected COVID-19 specimens, class II A2 or B2 BSCs have been recommended by the ICMR (Indian Council of Medical Research 2020; Mourya *et al.*, 2020). It has been observed that class II BSCs are generally preferred for work in BSL-2 laboratories. Whenever the question arises for the selection of an appropriate BSC for a containment facility, most of the users prefer the class II B2 BSC considering its 100% exhaust [Fig. 1(d)].

However, even though 100% of the air entering the BSC from the room and from the workspace is applied to the exhaust HEPA filter, there are chances of overloading the HEPA filter, whereas this difficulty does not arise in recirculation-type BSCs. In class II B1 BSCs, there is more than 50% exhaust and less than 50% recirculation of the air, and there is a shared boundary between the positive and negative pressure areas [World Health Organization (WHO), 2020c] [Fig. 1(c)]. Class II B2 BSCs have to be kept running all the time and, thus, require constant power supply in order to maintain negative pressure in the workspace. When the BSC is shut, contaminated air from the BSC might leak into the laboratory environment. In addition, as the exhaust is through the hard duct, these BSCs increase stress on the heating, ventilation, and air conditioning system and also create imbalance in the negative pressure of the laboratory. Installation of class II B1 or B2 cabinets needs to be done at the time of designing the laboratory. Thus, these factors need to be considered before selecting class II B1 or B2 BSCs.

In class II A1 and A2 BSCs, 30% of the air from the cabinet is exhausted in the laboratory environment, whereas 70% is recirculated in the BSC after filtration through the HEPA filter. In addition, class II A1 and A2 BSCs can be installed in the laboratory at any time. Even though the exhaust percentages of the class II A1 and A2 BSCs are the same, the class II A1 type BSC is not preferred as its common duct area is positively pressurized and chances of leakage of contaminated air in the laboratory through the gasket or joints exist [Fig. 1(a)]. These drawbacks have been overcome in the class II A2 BSC, where there is no proximity of the positive pressure duct to the areas under negative pressure containing potentially contaminated air [Fig. 1(b)]. It has been reported by Taylor *et al.* that the use of a class II A2 BSC for hazardous compounding in the pharmaceutical industry provides a comparable level of safety for the environment, users, and product while having less stringent airflow requirements as compared to a class II B2 BSC (Taylor *et al.*, 2019). Therefore, class II A2 BSCs may be preferred for work with COVID-19-related samples and SARC-CoV-2. In addition to this, the PPE acts as the last line of defense to the laboratory workers. Therefore, the use of appropriate PPE and its proper removal after completion of work, along with good laboratory practices, is necessary while handling infectious agents in all types of BSCs [World Health Organization (WHO), 2005].

## INSTALLATION, CERTIFICATION, AND DECONTAMINATION OF BSCs

Considering the infectious nature of the specimens being handled, it is important that the BSC is appropriately placed, installed, certified, and operated. For proper functioning of BSCs, the following installation requirements should be fulfilled. The BSC needs to be located far from the laboratory circulation zones in order to avoid air currents that could affect the curtain of air inside the cabinet. It is important to verify that the BSC is not installed alongside other types of cabinets such as chemical hoods. For factors such as an electrical connection equipped with the respective control and safety elements, an electrical outlet with earthing is required and the floor on which it is located should be flat and leveled, and the free space around the BSC recommended by the manufacturer needs to be respected. The ceiling of the room must be of recommended height so that the BSC can function without hindrance. After installation of the BSC, the installation, operational, and performance qualification parameters must be verified and fulfilled. The BSCs should be used as per the good laboratory practices (https://www.who.int/ihr/publications/biosafety-video-series/en/).

Considering the extent of morbidity and mortality caused by SARS-CoV-2, and other infectious agents that require handling inside a BSC, it is imperative to ensure that only certified BSCs should be used. The guidelines on the biosafety, construction, certification, and installation of BSCs are provided by international standards such as American Standard NSF 49, European Standard EN 12469, Australian Standard AS 2252, and Japanese Standard JIS K 3800. It is necessary to get the BSCs certified from accredited agencies or certified engineers to ensure whether all these protections are in place. Reports of certification should be maintained. Labels of certification should be displayed on the BSC for the identification of the status of certification. The user should monitor proper functioning of the BSCs by means of indicators. When equipment qualification is conducted by a standard endorsed third party such as an accredited agency for a particular division, the process is called certification. For the certification of BSCs, at least the following tests have to be carried out: inflow/face velocity testing, down flow velocity testing, airflow smoke pattern test, HEPA filter leak test, sash alarm test, and light intensity test (NSF/ANSI 49-2020, 2021).

The inflow/face velocity test confirms the performance of the blower and detects blockages in the HEPA filters, if any. The airflow smoke pattern test enables the visualization of the pattern of airflow inside the BSC, thereby giving an idea about the product and personal protection. The integrity testing of the HEPA filter confirms that there is no leakage in the filter and whether the filter has been fixed properly. After satisfactory results of all these tests are obtained, it can be confirmed that the BSC functions properly and provides all the required protection.

The certification of BSCs should be conducted at the time of installation, annually, after shifting the BSC from one place to another, and also after conducting major repair work such as replacement of the blower or filter. The use of BSCs containing a burn-out unit has not been recommended in any of the standard NSF/EN guidelines, and it is not required (NSF/ANSI 49-2020, 2021; BS EN 12469, 2000).

After the use of BSCs, it is necessary to ensure that it is decontaminated properly using disinfectants. Ethanol (62%–71%), sodium hypochlorite (1%) followed by a wipe down with 70% ethanol, 0.5% hydrogen peroxide, quaternary ammonium compounds, phenolic compounds, etc., have been recommended for use by the WHO (World Health Organization, 2020f). The ultraviolet (UV) light present inside the BSC can also be used to carry out surface decontamination every time before initiating work and after completion of work. If UV lamps are used, there needs to be additional care of ensuring that the lamps are clean and replaced after the recommended period of use. The decontamination of the BSC should also be done by fumigation using an appropriate method before opening the surfaces or internal components for carrying out any maintenance work, including but not limited to the following: changing filters, conducting tests requiring access to the interior surfaces or exposure of the cabinet, before conducting certification tests, before moving the BSC to a different location, or after the spill of a material containing high-risk agents.

## CONCLUSIONS

In the current scenario of the COVID-19 pandemic, class II A2 BSCs are a good choice over other types of BSCs in order to maintain biosafety and to safeguard public health. In addition to handling potentially infectious agents, there could be instances where the use of radio-isotopes or hazardous chemical research could be required. Therefore, a different BSC type may be recommended, based on risk assessment and continuous discussion between the biosafety representative and the investigators. The present study could be useful for the selection and use of BSCs in diagnostic and research laboratories that are handling or planning to handle SARS-CoV-2.

## Data Availability

Data sharing is not applicable to this article as no new data were created or analyzed in this study.
